# High acceptability of cognitive screening in HIV-infected patients: a pilot study

**DOI:** 10.7448/IAS.15.6.18099

**Published:** 2012-11-11

**Authors:** D Fasel, U Kunze, A Monsch, M Battegay

**Affiliations:** 1 University Hospital Basel, Clinic of Infectious Diseases, Basel, Switzerland; 2 University Hospital Basel, Memory Clinic, Basel, Switzerland

## Abstract

With combined antiretroviral therapy (cART) life expectancy of HIV-infected persons is close to the one of non-infected persons. Identifying neurocognitive deficits in ageing HIV-infected individuals is important. This study aimed to evaluate the acceptability of screening neurocognitive deficits in HIV-infected patients. Thirty patients (26 men, 4 women) from the HIV clinic were examined with a new screening test and an in-depth neuropsychological examination. The screening tests consisted of questions and examinations on cognition in everyday situations, mood and selected cognitive functions (word list memory, grooved pegboard, psychomotor speed, trail-making test, psychomotor speed and executive functions, digit symbol test). Also, patients received a questionnaire to evaluate test acceptance. The mean age of the patients was 52.5 (30–74) years, mean education 12.5 (8–18) years. Seven patients had HIV-stage CDC A, 12 B and 11 CDC stage C. The mean CD4 count was 657 cells/µl, the mean HIV viral load<20 cop./µl. All patients were treated with cART (7 with efavirenz). The screening test was done assisted by a nurse and lasted 26 minutes (mean). The screening indicated pathological signs of neurocognitive function in 11 (42%) patients. The in-depth neuropsychological assessment revealed pathological conditions in 25 (83%) of patients; i.e. 16 (53%) patients had ANI (asymptomatic neurocognitive impairment), 8 (27%) had MND (mild neurocognitive disorder) and 1 (3%) had HAD (HIV-associated dementia). Most patients (43.3%) judged the test as not too difficult and 56.6% as partly difficult. 96.6% of patients viewed the instructions of nurses as clear, 3.3% as unclear. 93.3% felt the test has not affected privacy and 83.3% estimated the screening as valuable and not worriesome. 83.4% of all patients were interested in their results and for none of the patients the test was too long. The test acceptability by the study nurses was also good. Only in 3.4% of tested persons they judged the test as too difficult for the patient. In 86.7% of tests they estimated the screening as valuable and in again 86.7% as not worrisome. For none of the nurses the test duration was too long. Only 16.6% of the patients had a completely normal neurocognitive testing. A short screening test lasting less than half an hour to search neurocognitive disorders assisted by a nurse is widely accepted by patients and nurses.

